# Decision-making in the diagnosis of tuberculous meningitis

**DOI:** 10.12688/wellcomeopenres.15611.1

**Published:** 2020-01-23

**Authors:** Tom H. Boyles, Lutgarde Lynen, James A. Seddon, Tuberculous Meningitis International Research Consortium

**Affiliations:** 1 ANOVA Health Institute, Johannesburg, South Africa; 2 Infectious and Tropical Diseases, London School of Hygiene & Tropical Medicine, London, UK; 3 Department of Clinical Sciences, Institute of Tropical Medicine, Antwerp, Belgium; 4 Department of Infectious Diseases, Imperial College London, London, UK; 5 Desmond Tutu TB Centre Department of Paediatrics and Child Health, Stellenbosch University, Stellenbosch, South Africa

**Keywords:** Tuberculosis Meningitis Diagnosis Threshold Decision

## Abstract

Tuberculous meningitis (TBM) is the most devastating form of tuberculosis (TB) but diagnosis is difficult and delays in initiating therapy increase mortality. All currently available tests are imperfect; culture of *Mycobacterium tuberculosis* from the cerebrospinal fluid (CSF) is considered the most accurate test but is often negative, even when disease is present, and takes too long to be useful for immediate decision making. Rapid tests that are frequently used are conventional Ziehl–Neelsen staining and nucleic acid amplification tests such as Xpert MTB/RIF and Xpert MTB/RIF Ultra. While positive results will often confirm the diagnosis, negative tests frequently provide insufficient evidence to withhold therapy. The conventional diagnostic approach is to determine the probability of TBM using experience and intuition, based on prevalence of TB, history, examination, analysis of basic blood and CSF parameters, imaging, and rapid test results. Treatment decisions may therefore be both variable and inaccurate, depend on the experience of the clinician, and requests for tests may be inappropriate. In this article we discuss the use of Bayes’ theorem and the threshold model of decision making as ways to improve testing and treatment decisions in TBM. Bayes’ theorem describes the process of converting the pre-test probability of disease to the post-test probability based on test results and the threshold model guides clinicians to make rational test and treatment decisions. We discuss the advantages and limitations of using these methods and suggest that new diagnostic strategies should ultimately be tested in randomised trials.

## Introduction

Tuberculous meningitis (TBM) is the most devastating form of tuberculosis (TB) with more than 100,000 new cases occurring each year ^1^. Most cases of TBM are not diagnosed and mortality without treatment approaches 100%. Outcomes are poor, even with treatment, particularly in children and patients who are co-infected with HIV. Of children who receive treatment, 20% die and over half of survivors have long-term neurodisability ^2^. For individuals living with HIV, TBM mortality is around 60%, even when treatment is initiated ^3– 5^. Delayed diagnosis and treatment are important risk factors for poor outcome and yet diagnosis can be difficult. Clinical and laboratory results commonly overlap with other diagnoses and cerebrospinal fluid (CSF) cell counts, glucose levels and protein levels are not diagnostic for TBM; but rather provide suggestive characteristics that indicate the diagnosis. These parameters can also be normal in up to 5% of culture-confirmed cases ^6^. Culture of *Mycobacterium tuberculosis* from the CSF occurs in around a half of adults, but is less common in children ^7^. While culture confirmation is an important part of the case definition of TBM ^8^, it is too slow to be of value in making immediate treatment decisions.

The most widely available rapid tests are smear microscopy on CSF following conventional Ziehl–Neelsen staining (CZN) and recently introduced nucleic acid amplification tests (NAATs) such as Xpert MTB/RIF and Xpert MTB/RIF Ultra (Ultra; Cepheid, Sunnyvale, CA, USA). The specificity of these tests is very high, and treatment should be started in almost all cases if positive. Sensitivity, however, is lower. The sensitivity of CZN is highly operator dependent, ranging from 12% to 47% at different sites in a recent study ^9^. The pooled sensitivity of Xpert MTB/RIF and Xpert MTB/RIF Ultra were 71% and 90% respectively, but were lower for patients with HIV (58% to 81%) ^10^. As a result, in many scenarios a negative test result is insufficient evidence to withhold therapy. There are no studies of Ultra in children with suspected TBM.

A new framework is required around decision-making in the diagnosis of TBM. Given that all existing tests are imperfect, it is important that we understand when we should be undertaking these tests, what these tests are able to tell us, and how they might influence our decision-making around starting treatment. We also need to use this framework to understand the place of new tests as they are developed and evaluated. In this article we discuss the use of Bayes’ Theorem, test parameters, such as likelihood ratios, and the concept of the *therapeutic threshold* to make better decisions in the field of TBM.

## Conventional approach to diagnosing TBM

The conventional diagnostic approach begins with a clinical evaluation of the patient, involving history, examination and analysis of basic blood and CSF parameters. In large clinical cohorts comparing features of patients with TBM to those without, several clinical factors are more common in those with TBM ( Table 1). These include young age, male sex, extrapyramidal movements, neck stiffness, longer duration of symptoms, focal neurological deficit (including cranial nerve palsy), higher temperature, and lower Glasgow Coma Scale score. HIV positivity, lower CD4 count, and lower serum sodium are also more common in patients with TBM than controls. Important CSF parameters that might help discriminate those with TBM from those without are CSF appearance, total leukocytes, total neutrophils, total lymphocytes, protein, glucose, Gram stain, adenosine deaminase activity, lactate dehydrogenase, India ink stain and cryptococcal antigen. Other potentially helpful tests are brain imaging and the search for extra-neural TB ^11^.

**Table 1.** Published clinical diagnostic rules for the diagnosis of tuberculous meningitis (TBM). CSF=cerebrospinal fluid (adapted from Wilkinson *et al.*) ^1^.

Publication	Population from which rule derived	Case comparison	Predictors of TBM	Area under receiver operator characteristic curve
Kumar *et al.* ^16^	Children from India	TBM versus other meningitis	Symptoms ≥7 days Optic atrophy Focal neurological deficit Extrapyramidal movements CSF leukocytes <50% neutrophils	Not reported
Thwaites *et al.* ^17^	Adults from Vietnam	TBM versus bacterial meningitis	Age <36 years Blood leukocytes <15 × 109/l Symptoms ≥6 days CSF leukocytes <750/mm3 CSF neutrophils <90%	0.99
Youssef *et al* ^18^	Children and adults from Egypt	TBM versus bacterial meningitis	Symptoms >5 days CSF leukocytes <1000/mm3 Clear CSF CSF lymphocytes >30% CSF protein >100 mg/l	Not reported
Cohen *et al.* ^19^	Adults from Malawi (77% HIV-infected)	TBM versus cryptococcal meningitis	Low CSF opening pressure Neck stiffness Raised CSF leukocytes Low Glasgow Coma Scale score High fever	0.87
Patel *et al.* ^20^	Adults from South Africa (84% HIV-infected)	TBM versus other meningitis	CSF:blood glucose ratio ≤0.2 CSF lymphocytes >200/mm3 CD4+ cell count <200 × 106/l Negative cryptococcal antigen test	Not reported
Hristea *et al.* ^21^	Adults from Turkey	TBM versus viral meningitis	Symptoms ≥5 days MRC grade II or III CSF:blood glucose ratio <0.5 CSF protein >100 mg/dl	0.977
Vibha *et al.* ^22^	Adults from India	TBM versus bacterial meningitis	Living in a rural area Symptoms ≥6 days Cranial nerve palsy Hemiplegia Clear CSF CSF neutrophils <75%	0.996
Dendane *et al.* ^23^	Adults from Morocco	TBM versus bacterial meningitis	Female sex Symptoms ≥10 days Focal neurological deficits Blood leukocytes <15 × 109/l Plasma sodium <130 mmol/l CSF leukocytes <400/mm3	0.906
Zhang *et al.* ^24^	Adults from China (all HIV-uninfected)	TBM versus cryptococcal meningitis	Female sex Reduced consciousness No visual or hearing loss Evidence of extraneural tuberculosis CSF leukocytes ≥68/mm3 CSF protein >0.91 mg/dl	0.931
Qamar *et al.* ^25^	Children from Pakistan	TBM versus bacterial meningitis	Hydrocephalus on brain CT CSF leukocytes <800/mm3 CSF protein:glucose ratio ≥2	0.90
Chusri *et al.* ^26^	Adults from Thailand with non-suppurative meningitis	TBM versus other meningitis	HIV infection Diabetes mellitus Symptoms <14 days Hydrocephalus CSF ADA level >10 IU	0.95
Lee S *et al.* ^11^	Adults from Korea	TBM versus viral meningitis	Serum sodium < 135 mmol/L CSF lactate dehydrogenase > 70 (U/L) CSF protein > 160 (mg/dL) Voiding difficulty Cranial nerve palsies	0.90

Typically, clinicians estimate a probability of TBM based on the evidence that is available to them using experience and intuition. In addition to the clinical factors mentioned above, this also includes factors such as the prevalence of TB in the geographical region that the patient may have been infected, and evidence of exposure through a close contact with TB. Inevitably therefore, there is significant variability in these estimates depending on the experience of clinicians. To standardise decision making, there have been multiple attempts to develop and validate multivariable prediction models to calculate the probability of TBM in a reproducible way. At least 12 models have been published with area under receiver operator characteristic curve ranging from 0.90 to 0.99, but a major limitation is that the performance of these models is variable when they are validated in new populations and settings ( Table 1). A major reason for this is case mix variation, meaning that the distribution of predictor variables such as HIV status, age, and the prevalence of TBM are variable. This can lead to differences in model performance, even when the value of predictors is consistent ^12^.

Conventionally, when new information in the form of test results or response to treatment becomes available, a clinician will update their estimate of the probability of TBM using experience and intuition. Ultimately, the clinician decides about the need to start, withhold, or stop TB therapy. Bayes’ theorem is a mathematical approach for the process of updating probabilities based on new information, which gives insight into the intuitive decision making of clinicians ^13^.

## Bayes’ theorem

**Figure 1.**
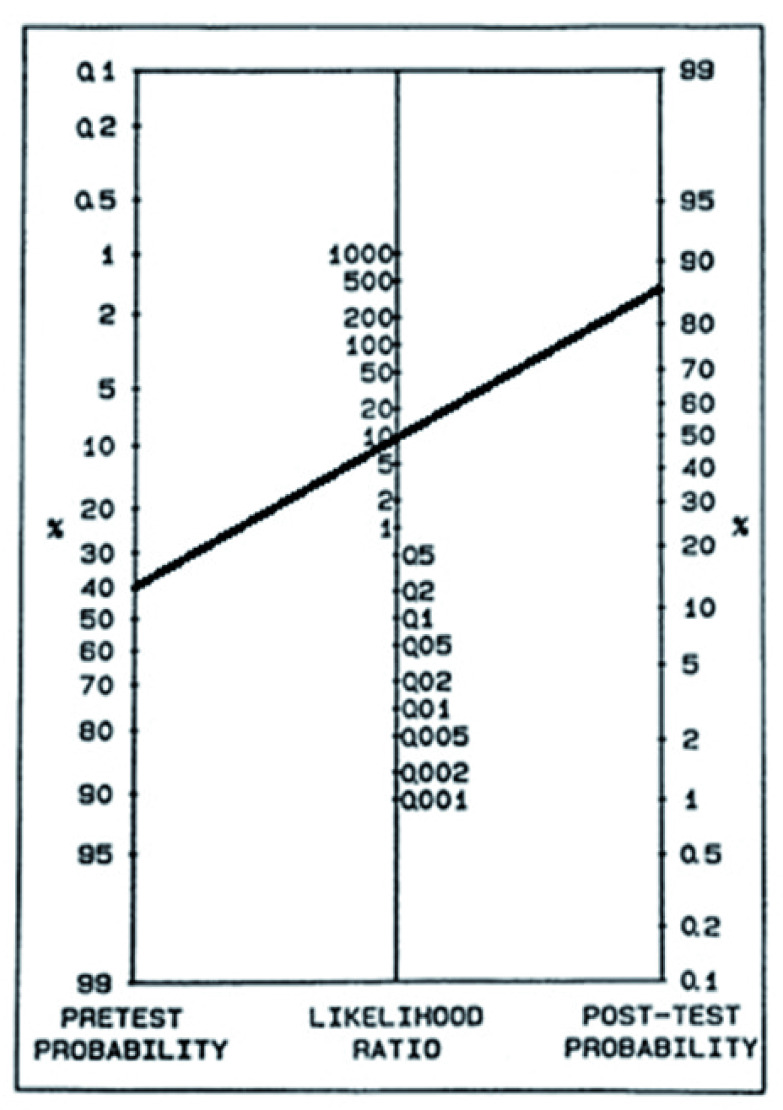
Fagan nomogram, the black line begins at pre-test probability (40%), travels through the positive likelihood ratio (10) to arrive at the post-test probability (85%). Values chosen for illustration only.

Bayes’ theorem describes the process of converting the pre-test probability of disease to the post-test probability based on new information becoming available, usually in the form of a test result. The information from the test is defined by the positive and negative likelihood ratios. The positive likelihood ratio is the probability of a person who has the disease testing positive, divided by the probability of a person who does not have the disease testing positive and ranges from 1 (no diagnostic value) to infinity (confirms the diagnosis when positive). The positive likelihood ratio can be calculated from a diagnostic accuracy study as sensitivity/ (1- specificity). The negative likelihood ratio is the probability of a person who has the disease testing negative divided by the probability of a person who does not have the disease testing negative and ranges from 0 (excludes the diagnosis when negative) to 1 (no diagnostic value). The negative likelihood ratio can be calculated from a diagnostic accuracy study as (1-sensitivity)/ specificity. Combining pre-test probability with the likelihood ratio gives the post-test probability, which is represented visually with a Fagan nomogram ( Figure 1) ^14, 15^.

## The threshold model of decision-making

Tests are only useful if the result is likely to influence patient management. The potential of a test to influence treatment decisions can be understood with the threshold model of decision making, which was first described in 1975 ^27^. Pauker and Kassirer first described the *therapeutic threshold*, which is the probability of disease at which the clinician feels indifference between treating or not treating a patient when no further diagnostic tests are available. It can also be defined as the probability at which the risks and benefits of treatment, such as drug toxicity, clinical cure, and the ability to stop other empiric therapies, balance the risks and benefits of no treatment, such as progression of untreated disease and the avoidance of drug toxicities. The *therapeutic threshold* can vary considerably from one condition to another and depends on the clinical condition of the patient, as the costs of withholding treatment from a severely ill patient are greater than in a less severely ill patient. It also varies depending on the subjective weight given to risks and benefits by individual clinicians and informed patients. It also changes over time if the patient’s condition improves or deteriorates.

There is no consensus on the optimal method of determining the *therapeutic threshold*, despite multiple attempts by investigators to quantify this parameter ^28– 32^. Only one attempt has been made to determine the therapeutic threshold for TBM. In this study, only adults with HIV co-infection were included and it was found that the *therapeutic threshold* values ranged from 51.4% for a very stable patient to 0% for a very unstable patient ^33^. This means that clinicians felt that if the patient was clinically stable and the probability of TBM was <51.4% when no further tests were available, they would prefer a watch and wait approach to initiating empiric TBM therapy. For a very unstable patient the probability would have to be close to zero before they would use this approach, preferring to offer empiric therapy in most cases.

In 1980, Pauker and Kassirer introduced two further thresholds based on the availability of a single diagnostic test with imperfect accuracy, a situation that is common in TBM, namely a *test-treatment threshold* and a *test threshold* ^32^. Most relevant to TBM is the *test-treatment threshold*, which is the point of equipoise regarding the decision to gather additional data by doing the test or to rule in the disease and initiate treatment without the need for a test. When the pre-test probability lies above *test-treatment threshold*, the post-test probability will lie above the *therapeutic threshold* even if the test is negative and so the test will not influence the decision to initiate therapy.

**Figure 2.**
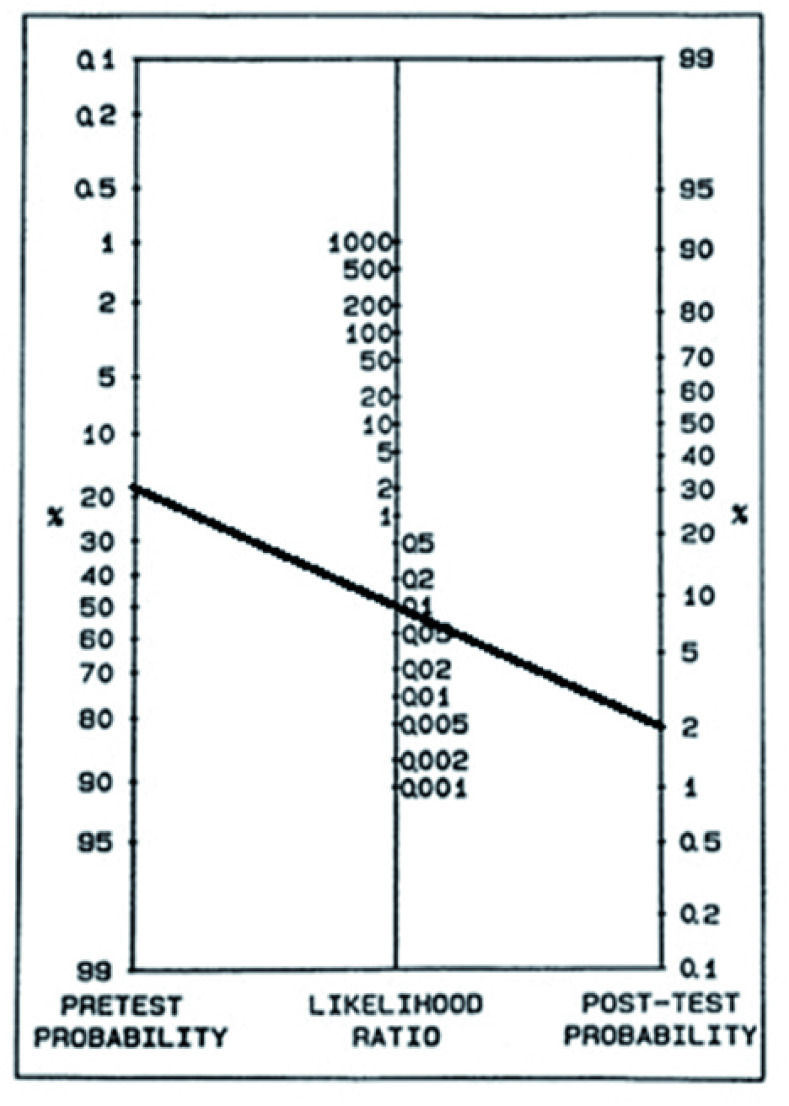
Fagan nomogram illustrating a realistic tuberculous meningitis scenario. The therapeutic threshold (post-test probability) is 2% and negative likelihood ratio 0.01. The pre-test probability is 20% which defines the test-treatment threshold as any pre-test probability greater than 20% will give a post-test probability above 2% and will not influence the decision to initiate therapy.

**Figure 3.**
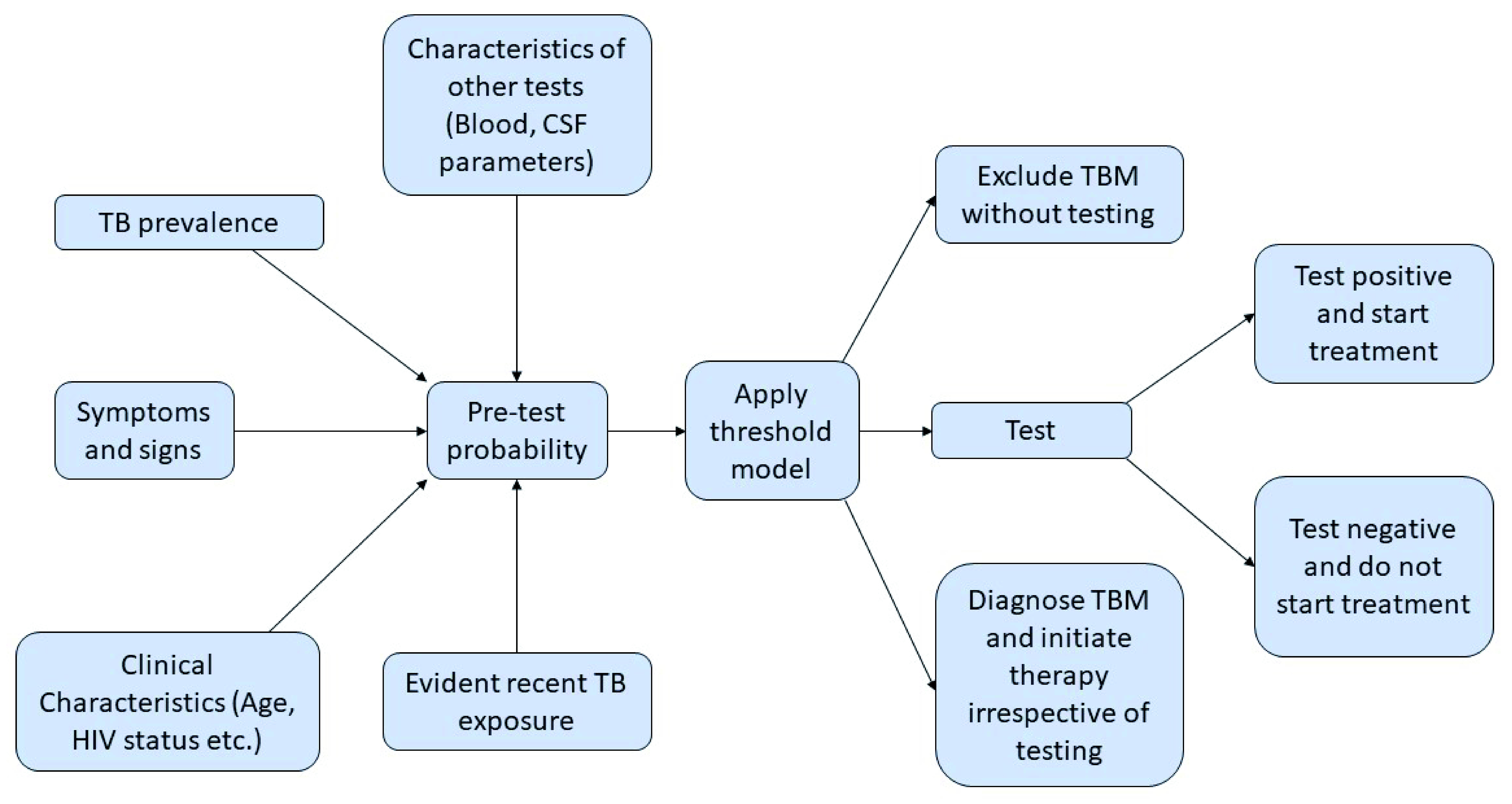
Using a threshold model to make decisions on whether to use a diagnostic test. TBM -Tuberculous meningitis, CSF – Cerebral spinal fluid.

This is illustrated in Figure 2. In this example, the *therapeutic threshold* and negative likelihood ratio have been determined empirically to be 2% and 0.1 respectively. The line from 2% post-test probability (equated to the *therapeutic threshold* in our example), passes through the likelihood ratio of 0.1 and intersects the pre-test probability at 20%. This defines the *test-treatment threshold*, as at any pre-test probability greater than 20%, a negative test will give a post-test probability above 2%, and treatment should be started regardless of the result. It follows that for any given *therapeutic threshold* and negative likelihood ratio, the *test-treatment threshold* can be recalculated using Bayes’ theorem. The *test-treatment threshold* is an important concept in decision-making for TBM and other diseases. Testing will sometimes be appropriate even when the pre-test probability is above the *test-treatment threshold*, for example to determine whether other diagnoses should be pursued or to detect drug resistance, but it is important that treatment is initiated even if the test is negative. ( Figure 3).

Pauker and Kassirer defined the *test threshold* as the point of equipoise regarding the decision to rule out the disease or gather additional data by performing a test ^32^. For TBM, the *test threshold* is of less relevance when CZN or Ultra are available as any positive test will mean that treatment should be initiated. In practice, the *test threshold* for TBM is influenced largely by the availability of resources. Use of the threshold model of decision-making has the potential to improve treatment decisions and reduce the costs associated with unnecessary tests, which can be financial or clinical. It also reduces the likelihood of clinical errors occurring, such as a clinician being unduly influenced by a negative Xpert Ultra and withholding TB treatment, when the probability of disease remains above *therapeutic threshold*. However, the model is based on several assumptions that are not always met and has important limitations.

**Figure 4.**
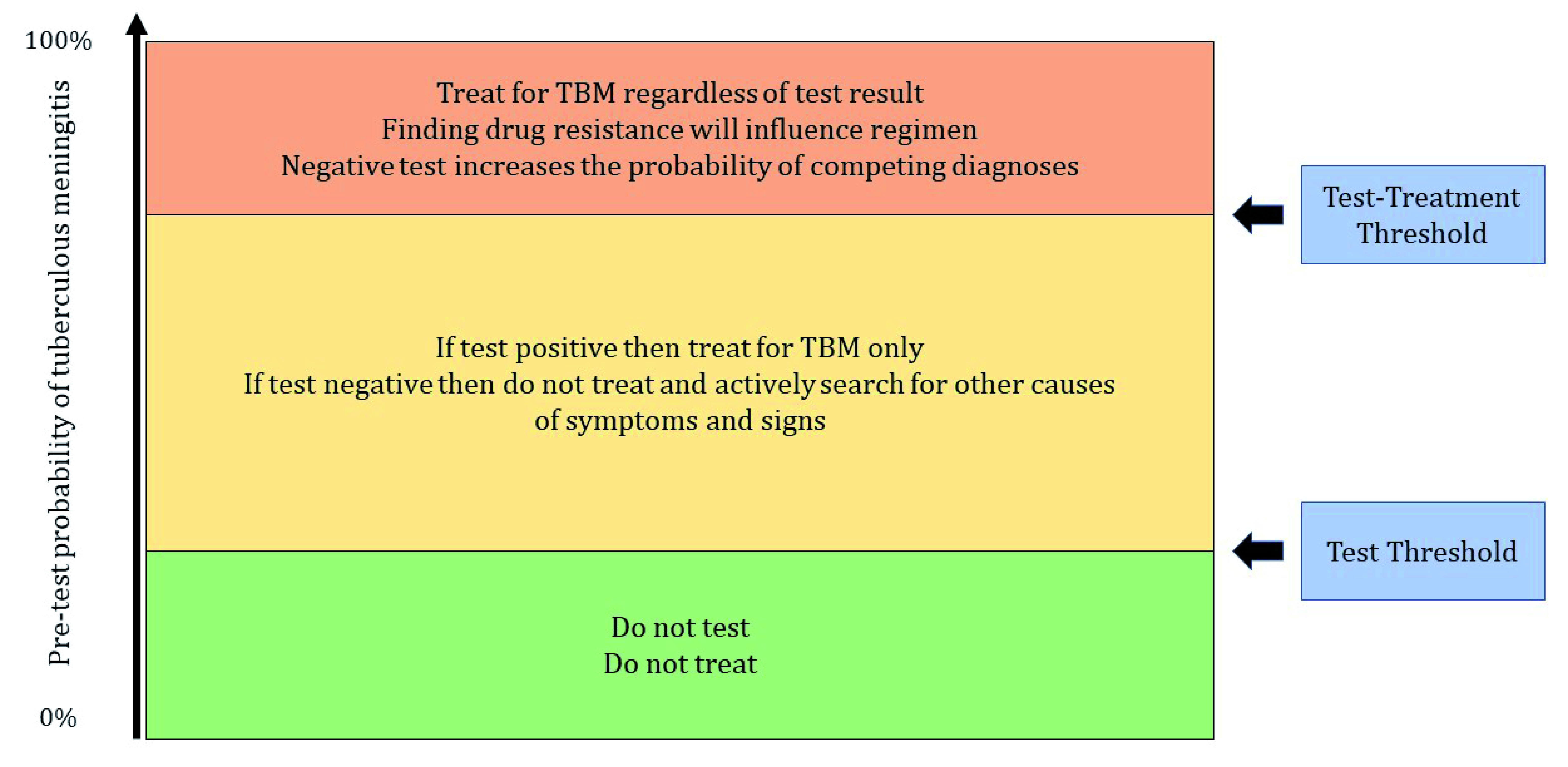
A visual depiction of how pre-test probability and both the test threshold and test-treatment threshold influence clinical decision making. TBM -Tuberculous meningitis.

One limitation is that it relates to a single cause for the patient’s symptoms, with a single definitive treatment. The original example given by Pauker and Kassirer was a patient with right iliac fossa pain who may or may not require an operation for appendicitis, with an implicit assumption that a cause of the symptoms other than appendicitis was of limited clinical significance ^27^. However, patients with clinical features in keeping with TBM often have several important competing diagnoses, such as cryptococcal, viral, or bacterial meningitis, which complicate decision-making. So, while a positive or negative test result may not influence the decision to initiate treatment for TBM, it may influence the probability of competing diagnoses. For example, a positive CZN or Ultra may allow treatments for alternative conditions, such as antibiotics to be confidently withheld. Similarly, a negative test for TBM may not mean that TB treatment is withheld, but it may increase the probability of alternative diagnoses and therefore prompt further testing or empiric treatments ( Figure 4). It is also possible that a positive test may result in the initiation of treatment at an earlier stage than if the diagnosis is not confirmed. For TBM, any delay in treatment initiation can have severe consequences.

Complexity also arises with the use of NAATs that answer more than one question. For example, Xpert Ultra sometimes provide additional useful information about the presence of rifampicin resistance, which has important implications for the choice of drug regimen. It could be argued that Ultra should be performed regardless of the pre-test probability of TBM, as a finding of rifampicin resistance will always influence patient management. In practice, the decision to use Ultra purely as a test for rifampicin resistance should be based on local prevalence, patient factors such as previous TB treatment, and availability of resources.

A final aspect of clinical care, not addressed by the threshold model of clinical decision-making, is the inherent value to patients, clinicians and researchers in confirming a diagnosis. For patients, a confirmed test result may increase confidence in the diagnosis, and consequently their willingness to complete long and sometimes unpleasant treatment. For clinicians, a confirmed diagnosis reduces the time, effort, risk and cost associated with investigating for other causes of the symptoms and increases the confidence that the patient has in the ability of the clinician, likely improving the doctor-patient relationship. Finally, for epidemiological surveillance and both diagnostic and therapeutic research, a confirmed diagnosis increases the rigour of the scientific question being evaluated.

## Everyday use of Bayes’ theorem and the threshold model of decision-making

To apply Bayes’ theorem and the threshold model of decision-making to TBM it is first necessary to develop validated clinical prediction models so that pre-test probability can be accurately determined from readily available data. A systematic review and individual patient data meta-analysis is currently underway to develop a prediction model that is generalizable to multiple geographical locations and different case mixes ^34^.

Next, it is necessary to determine the *therapeutic threshold* over the full range of patient disease severities, in children as well as adults, and in different geographical regions. Several methods have been used, prescriptive (based on calculations) or descriptive (derived from observing clinical practice). Prescriptive methods can be based purely on values of costs and benefits from the literature (e.g. expected utility theory) ^27, 32^ or include subjective values and socio-economic factors (e.g. regret based models and dual processing threshold models) ^30^. Descriptive methods rely on decision-making by clinicians when faced with clinical scenarios (e.g. derived thresholds and discrete choice experiments) ^28, 29^. All methods have strengths and weaknesses and there is no consensus on the best approach. It may be that the optimal strategy is to combine multiple different methods. 

Once the threshold has been established, diagnostic accuracy, in particular the likelihood ratios of available tests, needs to be determined using robust methods and be reported according to the STARD (Standards for the Reporting of Diagnostic Accuracy Studies) guideline ^35^. By combining the *therapeutic threshold*, negative likelihood ratios and pre-test probability it is possible to determine whether testing is necessary or whether empiric therapy should be offered.

An advantage of using Bayes’ theorem and decision thresholds is that once the *therapeutic threshold* has been determined empirically, it is possible to recalculate *test threshold* and *test-treatment threshold* whenever a novel test has been developed, based on the likelihood ratios. In this way, it is possible to determine the likely impact of a novel test in terms of the proportion of patients in which it would be likely to influence treatment decisions.

## Testing diagnostic strategies

While the use of the methods described above can theoretically improve treatment decisions and allow for the evaluation of novel tests based on diagnostic accuracy studies, it is important that diagnostic strategies are tested in real world randomised controlled trials ^36^. As an example, for a diagnostic strategy based on a clinical prediction model, the accuracy of a test and decision thresholds could be integrated into a smartphone application which gives advice to clinicians regarding the need to initiate empiric therapy, proceed to testing or hold off any testing or treatment. Such a strategy should be tested against the current standard of care in a randomised trial, possibly using a stepped wedge design, to determine if it can reduce mortality, morbidity, and costs.

## Conclusion

The consequences of withholding therapy from a patient with TBM is almost certain death and so treatment decisions can be highly emotive for both clinicians and patients. Currently, we have only a basic understanding of the best predictors of TBM from history, examination, and CSF interpretation, particularly in children and HIV-positive patients who are the most vulnerable. While the introduction and widespread use of Ultra would be welcomed, the imperfect sensitivity means that treatment should be started in patients with a moderate pre-test probability, regardless of the test result. Even carrying out the test should be queried if treatment will be started irrespective of test result. While the use of Bayes’ theorem and clinical decision thresholds has limitations, a clear understanding is key to evaluating existing and novel diagnostic strategies for TBM.

## Data availability

### Underlying data

No data are associated with this article
