# Supplementary material for: Decision-making in the diagnosis of tuberculous meningitis
Source: Wellcome Open Res. 2020 Jan 23;5:11. [Version 1] doi: 10.12688/wellcomeopenres.15611.1 (PMC7490569; doi:10.12688/wellcomeopenres.15611.1)
Supplement: Peer review history

# Peer review: Reviewer response for version 1

**DOI:** 10.21956/wellcomeopenres.17101.r40095

**Published:** 2020-09-23

**Authors:** Zongde Zhang

**Affiliations:** 1 Laboratory for Drug Resistant Tuberculosis Research, Beijing Chest Hospital, Capital Medical University, Beijing, China

Tuberculous meningitis (TBM) is the most devastating form of tuberculosis and is difficult to diagnosis. Conventional tests such as Ziehl–Neelsen staining and Culture of Mycobacterium tuberculosis from the cerebrospinal fluid (CSF) is considered the most accurate test but is often negative. The positive rate of newly developed nucleic acid amplification tests such as Xpert MTB/RIF and Xpert MTB/RIF Ultra is still low.

 The conventional diagnostic approach is to determine the probability of TBM using experience and intuition, based on prevalence of TB, history, examination, analysis of basic blood and CSF parameters, imaging, and rapid test results. Treatment decisions may therefore be both variable and inaccurate.

 This article discusses the use of Bayes’ theorem and the threshold model of decision

 making as ways to improve testing and treatment decisions in TBM. It will guide clinicians to make rational test and treatment decisions.

 One minor suggestion: Could the authors please use Bayes’ theorem to give an example by parameters of a TBM patient for a decision making?

Is the review written in accessible language?

Yes

Are all factual statements correct and adequately supported by citations?

Yes

Are the conclusions drawn appropriate in the context of the current research literature?

Yes

Is the topic of the review discussed comprehensively in the context of the current literature?

Yes

Reviewer Expertise:

Tuberculosis

I confirm that I have read this submission and believe that I have an appropriate level of expertise to confirm that it is of an acceptable scientific standard.

# Peer review: Reviewer response for version 1

**DOI:** 10.21956/wellcomeopenres.17101.r40094

**Published:** 2020-09-14

**Authors:** James J.C. Nuttall

**Affiliations:** 1 Paediatric Infectious Diseases Unit, Red Cross War Memorial Children's Hospital and Department of Paediatrics and Child Health, University of Cape Town, Cape Town, South Africa

This article describes clinicians’ decision-making processes about the diagnosis and initiation of treatment in patients with suspected tuberculous meningitis (TBM). The authors outline a mathematical framework based on understanding pre-and post-test probability and the threshold model of decision-making with the aim of making decision-making less subjective and more standardized. This is of particular relevance to TBM as microbiological confirmation of the diagnosis is constrained by the relatively low sensitivity of currently available tests, the delay to obtaining a culture result or lack of access to testing. 

 The therapeutic threshold for initiating TBM treatment in certain populations, such as children and HIV-infected patients in TB endemic regions, is appropriately low and the diagnostic utility of novel tests need to be critically evaluated in this context. An important contribution of the article is in highlighting how the pre-test probability of a diagnosis based on epidemiology and basic clinical and laboratory assessment impacts the interpretation of test results and the post-test probability of the diagnosis. The interpretation of negative tests results with low sensitivity should lead us to question whether or not the additional tests (for example nucleic acid amplification tests (NAAT)) are routinely justified as they may not alter clinical management and negative results have the potential to delay or miss a diagnosis of TBM resulting in treatment delays or inappropriate discontinuation of treatment. An acknowledged exception to a rigid test-treatment threshold approach includes the utility of positive NAAT tests not just in confirming a diagnosis of TBM but in rapid identification of drug-resistant TB in regions with high prevalence of rifampicin resistance and taking into account individual patient factors. 

 The variability in the performance of clinical prediction scoring systems in settings with different case mixes is also highlighted. Lack of well-validated pre-test probability estimates based on clinical information is currently a limitation of using the test threshold and test-treatment threshold approaches. Although the authors reasonably assert that validated clinical prediction models and therapeutic thresholds must be developed before Bayes’ theorem and threshold model of decision-making may be applied, this may be challenging in practice. Variability of TBM clinical presentations across geographical regions, age of patients, co-morbidities, clinical stage of disease at presentation, and the complexities of clinician experience and conscious or sub-conscious decision-making question whether or not a mathematically derived algorithmic approach will perform more reliably than conventional clinician-centred approaches.

 The article is well-written and clearly expressed with useful figures and tables. I have no additional specific comments.

Is the review written in accessible language?

Yes

Are all factual statements correct and adequately supported by citations?

Yes

Are the conclusions drawn appropriate in the context of the current research literature?

Yes

Is the topic of the review discussed comprehensively in the context of the current literature?

Yes

Reviewer Expertise:

Paediatric HIV and TB

I confirm that I have read this submission and believe that I have an appropriate level of expertise to confirm that it is of an acceptable scientific standard.

# Peer review: Reviewer response for version 1

**DOI:** 10.21956/wellcomeopenres.17101.r39828

**Published:** 2020-08-17

**Authors:** David W. Dowdy

**Affiliations:** 1 Department of Epidemiology, Johns Hopkins Bloomberg School of Public Health, Baltimore, MD, USA

This manuscript describes the application of Bayes' theorem and likelihood ratios to decision-making in the context of tuberculous meningitis (TBM). In my opinion, the manuscript is well written and makes its primary points clearly. I have a number of minor suggestions, as detailed below, but nothing that would require major revision on the part of the authors. Congratulations to the authors on putting together such a clear description of decision-making in the diagnosis of TBM.

1. It could be useful for the authors to actually write out Bayes' theorem. This manuscript is written for a level of readership that could likely understand the theorem and its application.
2. In the first paragraph under "Bayes' theorem" subheading, some of the language is a little imprecise. The likelihood ratio, for example, is a ratio of likelihoods, not probabilities - and these likelihoods are not "of a person who has the disease testing positive", for example. Likelihoods could be described in terms of probabilities, but it's probably useful not to conflate the two.
3. I don't feel strongly about this, but the authors might want to describe Bayes' theorem in terms of pre-test and post-test odds, so that readers without access to the Fagan nomogram could still make the relevant calculations. (Again, I don't feel strongly, and recognize that this could over-complicate the manuscript.)
4. Figure 3 could probably be clarified further, illustrating the test threshold, the test-treatment threshold, and the test result itself. Many of the boxes to the left (feeding into "pre-test probability") could likely be combined into a single box with multiple bullets. Then, for example, after "apply threshold model", indications could be given as to which box corresponds to a pre-test probability below the test threshold, between the test threshold and test-treatment threshold, and above the test-treatment threshold. Similarly, after "test", indications could be more clear about the result of the test, separating the test result from the treatment decision. This could be done by placing labels on the corresponding lines or using boxes of a different shape, for example.
5. The authors argue that the test threshold is less relevant for TBM - but there are still a large number of patients for whom the pre-test probability of TBM is sufficiently low that testing for TBM is never even considered by the clinician. This might be clarified in the manuscript - it's not that the test threshold is irrelevant, but rather that virtually all patients in whom TBM might ever be considered are likely to have a pre-test probability that falls above that threshold.
6. On p.6, line 10 of column 2, perhaps use an example other than "such as antibiotics" - since antibiotics would be used for TBM as well. ("Antifungal agents", perhaps?)
7. The authors implicitly assume that all Xpert testing is via Xpert Ultra cartridges (for example, "Xpert Ultra sometimes provide*s* additional useful information about the presence of rifampicin resistance"). Many of these statements are true of the earlier Xpert MTB/RIF cartridge as well - it may be worthwhile simply using "Xpert" rather than "Xpert Ultra" for these statements.
8. In Figure 4, consider changing the y-axis to more appropriately reflect the actual values of the thresholds under consideration. For example, the authors argue that the test-treatment threshold might reasonably be at a pre-test probability of 0.2 - but in this figure, it looks more like it is at 0.8.
9. In the section on "everyday use of Bayes' theorem...", the authors occasionally write as if a single threshold value can be established. Their nuanced description of different methods for setting thresholds is welcome, but they then proceed to suggest that a threshold can be established. At a minimum, they should acknowledge that this threshold may be different in different settings. But in reality, this threshold may vary at an individual patient or clinician level - and it may be worthwhile (briefly) discussing the pros and cons of this situation, relative to one in which a single agreed-upon threshold could be established.

Again, I would like to congratulate the authors on this important and well-written contribution, which reflects a tremendous amount of work. It has been a pleasure to review this piece, and I hope these comments are helpful.

Is the review written in accessible language?

Yes

Are all factual statements correct and adequately supported by citations?

Yes

Are the conclusions drawn appropriate in the context of the current research literature?

Yes

Is the topic of the review discussed comprehensively in the context of the current literature?

Yes

Reviewer Expertise:

epidemiology of tuberculosis

I confirm that I have read this submission and believe that I have an appropriate level of expertise to confirm that it is of an acceptable scientific standard.
